# Paint booth waste as an alternative aggregate for the production of interlocking concrete blocks

**DOI:** 10.1038/s41598-024-53668-y

**Published:** 2024-02-07

**Authors:** Catarina Monteiro Câmara, Suéllen Tonatto Ferrazzo, William Mateus Kubiaki Levandoski, Cristina Vitorino da Silva, Eduardo Pavan Korf

**Affiliations:** 1 Graduate Program in Environmental Science and Technology - PPGCTA, Federal University of Fronteira Sul, Erechim, RS 99700-970 Brazil; 2 Graduate Program in Civil Engineering, Federal University of Rio Grande do Sul - PPGEC, Porto Alegre, RS 90035-190 Brazil; 3 Undergraduate Program in Civil Engineering, Integrated Regional University of Alto Uruguay and Missions - URI, Erechim, RS 99709-910 Brazil

**Keywords:** Environmental sciences, Engineering

## Abstract

Inadequate disposal of hazardous waste results risks to the environment and human health. Although the use of hazardous waste in new processes and/or products has received limited attention in the literature, there is still significant potential to be investigated. Reducing the usage of natural resources and waste management are important for sustainable practices during concrete production. This study investigated the mechanical and leaching behavior of paint booth waste (PBW) as a partial substitute (10, 20, 30 and 40%) of coarse aggregate in concrete mixtures for the manufacture of interlocking blocks. A sample of PBW used in this research differs from those in the literature due to its granulometry characterized by aggregates of different sizes. Concrete consistency, compressive strength, water absorption, X-ray diffraction, scanning electron microscopy, and leaching tests were carried out. The PBW did not influence the consistency in the fresh state of the concrete. The blocks with smaller substitutions (10 and 20%) presented denser structures and with greater strengths, surpassing 35 MPa after 28 days. Higher levels of PBW resulted in more porous concrete blocks with greater water absorption. The concrete-PBW mixtures showed no metal toxicity, i.e., the incorporation of this waste in the construction material avoided metal leaching. Concrete blocks with up to 20% PBW demonstrated satisfactory mechanical and environmental performance.

## Introduction

Steel is one of the most used raw materials in the world and the painting of its parts is a process present in manufacturing to ensure corrosion control^1^. The use of epoxy paint has been a low cost alternative by the metallurgical industry due to its low cost and the efficiency in protecting parts against oxidation^2^. However, an organic solvent-based paint application is a serious environmental problem due to the amount of toxic substances in its formulation^3–5^. Although organic water-based paints and composite paints for minor organic volatiles (VOCs) have been developed, the industries opt for the organic solvent-based paints due to both its better finish and quick drying.

In these industrial processes, mechanical or manual overspray that does not adhere to the parts gives rise to paint booth waste (PBW). This waste consists of a complex material due to the presence of polymeric resins, surfactants, pigments and curing agents, making its reuse in industry unfeasible, making it a challenge for recycling. European Union regulamentation, for example, does not allow disposal of PBW in landfills, to encourage technologies to add value and reduce impacts^4^. Besides, studies to improve disposal logistics and minimize the impacts of this waste have been investigated in Canada^3^. New Zealand’s Green Building Council promotes more sustainable construction, encourages carbon reduction and the use of waste the in materials. To help receive the title of “world leadership” in building sustainability in this country, researches into paint waste on concrete has been studied more and more to reduce incineration these waste^12^. In Brazil it is estimated that in 2019 approximately 29,000 tons of hazardous waste were generated, 2323 tons of PBW being generated from the metallurgical^6^. As PBW is classified as a hazardous waste by^7^, it is normally sent to an appropriate destination for hazardous waste management, but due to its high costs and the usage of large areas for it, it is not considered a sustainable waste management solution.

An interesting alternative for hazardous waste (e.g. PBW) is through solidification/stabilization. These processes are carried out with the use of cementing agents, such as Portland cement and cement with the addition of pozzolanic material, making it possible to encapsulate the contaminants and reduce the leaching of heavy metals and organic pollutants^8,9^. In this context, PBW (in the form of sludge) has been studied as a partial replacement of lime and sand in mortar mixtures^5^, in the incorporation of red ceramic^10^ and in bituminous mixtures^11^ and also as a polymeric additive in concrete mixtures^12^.

On the other side of the system, there is the problem of using products that consume many non-renewable resources and release a high load of pollutants into the environment, such as concrete. After water, concrete is the second most used material in the world and it is estimated that around three tons of concrete is produced per person annually^13^. In addition to consuming a high amount of aggregates (i.e. approximately 40 billion tons per year), the production of concrete is responsible for about 4 to 8% of CO_2_ emissions, apart from other impacts caused during the production process^14^.

In this sense, several studies have investigated the incorporation of different types of waste as aggregate substitutes such as: recycled tire rubber^15^; iron ore waste^16^; textile sludge^17^; recycled aggregate with the addition of steel fiber^18^; waste coconut shell^19^ and plastic waste^20^. The use of alternative cements, such as application in conventional concrete mixtures and interlocking blocks, has also been the subject of study^12,21,22^.

**Figure 1:**
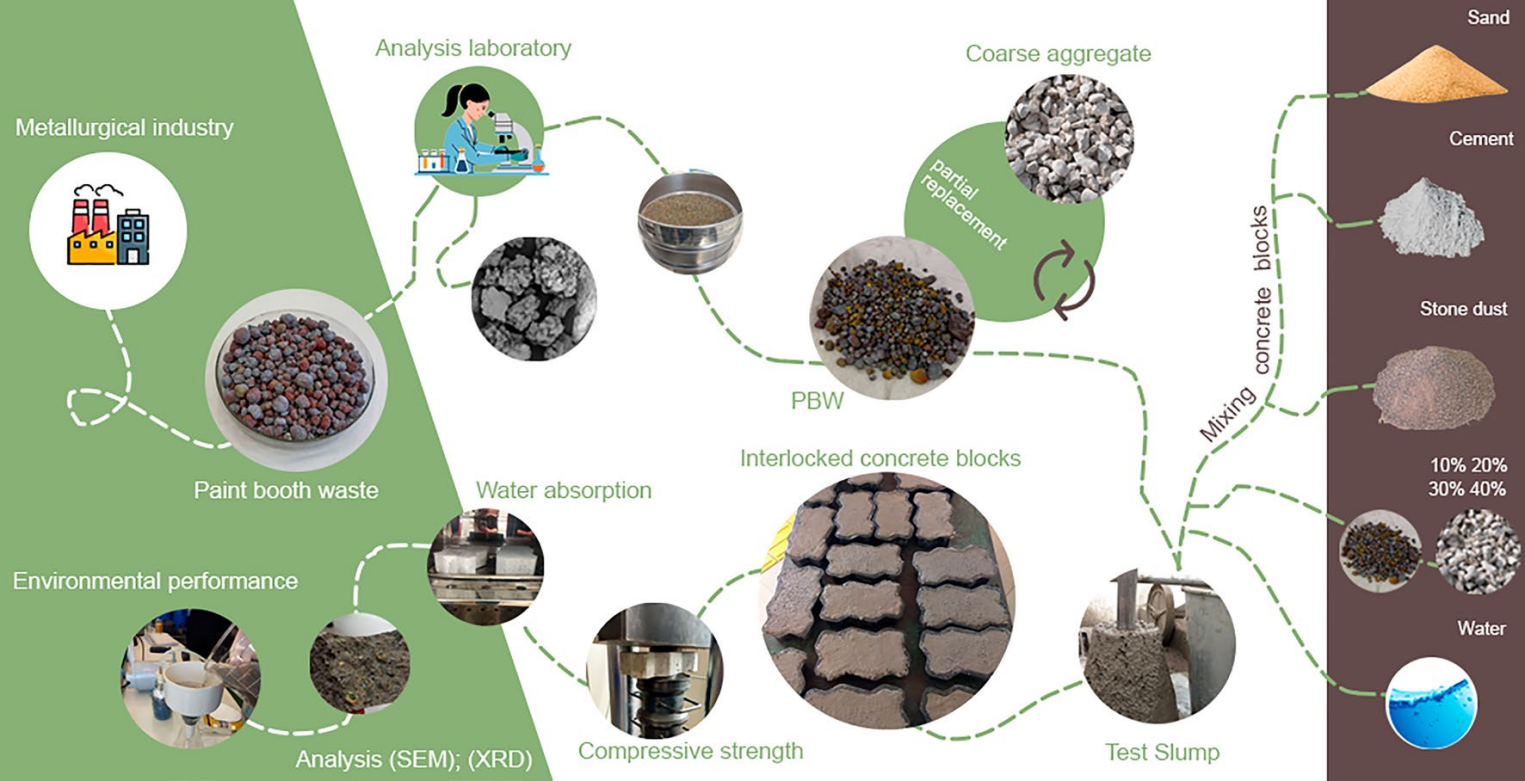
Schematization of this research.

Although the PBW of this study has chemical and mineralogical characteristics similar to those characterized by other researches^8,10,11,23^, here are no reports in the literature of investigations about their peculiar granulometry (i.e. they have a portion of fine material and aggregates of different sizes). In addition, it was identified that the use of this hazardous waste with joint investigation of environmental performance tests, in concrete mixtures represents a field of research still to be explored. Seeking to advance in this knowledge gap, this study evaluated paint booth wastes (irregular granulometry) as a partial substitute for coarse aggregate in concrete mixes, for interlocking paving (Fig. 1). The evaluation of the mechanical and environmental performance comprised PBW concrete consistency, strength and water absorption, as well as mineralogy, microstructure and metal leaching.

## Materials and methods

### Materials

High initial strength and sulfate-resistant Portland cement, specified in Brazil as CPV-ARI RS, was used. Based on the Brazilian standards^24,25^, both a density of 2.79 g/cm^3^ and a fineness modulus of 1.25% were determined.

The following were used as fine aggregates: natural river sand and stone dust. Besides that, coarse aggregate of basaltic origin was also used, as well as PBW as its substitute. The physical characteristics of these materials and respective determination rules are presented in Table 1. The shape index shows that the PBW has more spherical particles than the coarse aggregate, due to its values being closer to the unitary value. The lower density of PBW in relation to the aggregates reflects a more microporous material and also a lower presence of higher density minerals.

**Table 1:** Physical characteristics of aggregates and PBW for concrete mixing.

Aggregates	Density (g/cm^3^)	Reference standards	Maximum diameter (mm)	Fineness modulus	Reference standards	Shape index	Reference standards
Sand	2.30	NBR 16,916/21	2.36	2.50	NBR NM 248/03	–	–
Stone dust	2.40		4.75	3.39			
Coarse aggregate	2.75	NBR 16,917/21	9.75	5.74		2.02	NBR 7809/05
PBW	1.89	Gas pycnometer	19	5.89		1.21	

**Figure 2:**
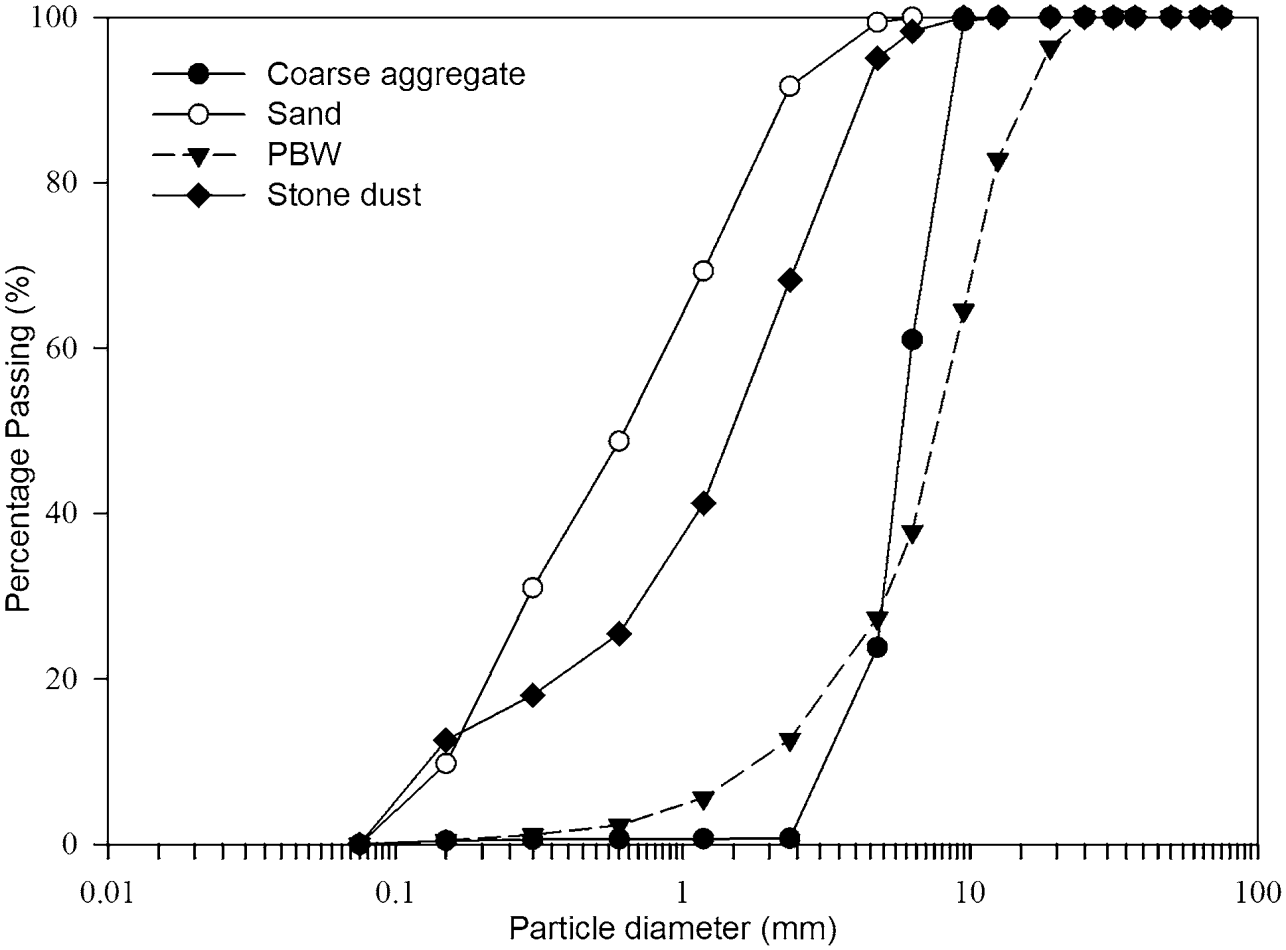
Particle size distribution of materials.

Figure 2 shows the particle size distribution curve for aggregates and PBW. From the curve it is possible to observe that the PBW has a granulometric characteristic more distributed than the coarse aggregate, as well as a greater amount of fine particles, which is corroborated by the higher fineness modulus and may be favorable for the packaging of the microstructure when it was used.

**Figure 3:**
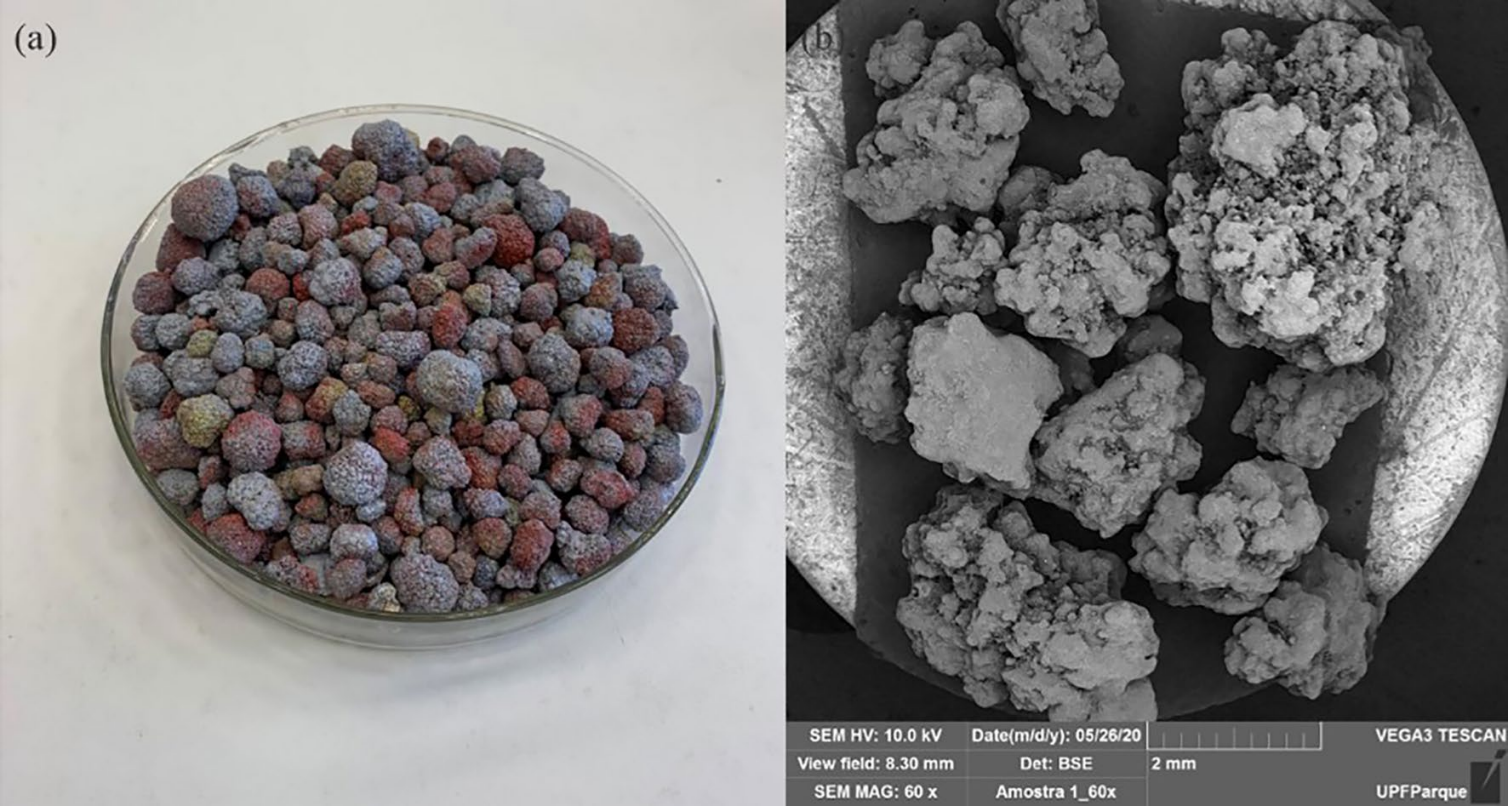
(**a**) Paint booth waste (PBW), (**b**) MEV of PBW 60 × magnification.

Paint Booth Waste (PBW) (Fig. 3a) comes from the metallurgical industry, located in the northern region of the state of Rio Grande do Sul in Brazil. Its density (Table 1) was projected from a helium gas pycnometer, using a Quantachrome Instruments pycnometer, model Ultrapycnometer 1200.

The mineralogy of the tailings was evaluated by X-ray diffraction (XRD), using the powder method, in an X-ray diffractometer (Bruker, model D8 Endeavor) with a position detector. The samples were scanned at two θ values from 2.5 to 70° with a step size of 0.02°, operated at 40 kV and 35 mA. The chemical composition was evaluated by means of X-ray fluorescence spectrometry (XRF), and the conditions were by means of tablets pressed in the STD-1 calibration (Standarless), without patterns of chemical elements between fluorine and uranium, in an X-ray fluorescence spectrometer (Malvern Panalytical, model Zetium).

**Figure 4:**
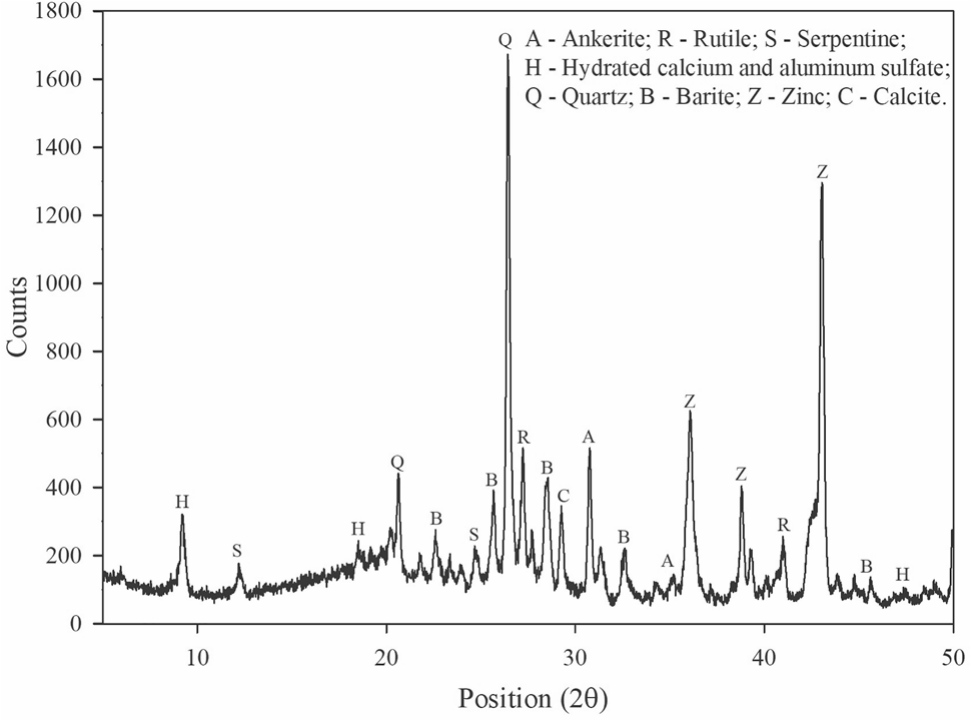
PBW diffractogram.

Mineralogical analyses (Fig. 4) demonstrate the predominance of calcium and magnesium carbonate minerals, such as calcite and ankerite; barium sulfate, such as the barite, in addition to titanium dioxide, such as the rutile. The chemical analysis identified a predominance of 17.6% of silicon dioxide (SiO_2_), 8.11% of barium oxide (BaO), 5.87% of aluminum chloride (TiO_2_) and 5.36% of zinc (ZnO), in addition to 45.9% loss on fire. In this study, paint waste was used as part of the coarse aggregate, using all the material that passed through the 12.5 mm sieve.

The PBW waste classification was evaluated^7^ and according to the Annex A of the aforementioned standard, the PBW is classified as hazardous waste, depending on its origin and nature. This is because PBW belongs to the class of “waste and sludge from paints from industrial painting”, with hazardous constituents (e.g. cadmium, chromium, lead, cyanide, toluene and tetrachlorethylene) that give it a “toxic” hazardous characteristic.

The superplasticizer (ADVA brand, FLOW 755) was used with a polycarboxylate-based additive for concrete with high water-reducing power, and a density between 1.015 and 1.055 g/cm^3^.

### Mixing and molding of interlocking concrete blocks

The proportion of materials used for the dosage of concrete is shown in Table 2. An adaptation of the method^15^, changing only the amount of stone dust and superplasticizer additive was performed.

**Table 2:** Portion of materials for measurements of the mixture of intervened concrete blocks.

Replacement content (%)	Cement	Sand	Stone dust	Coarse aggregate	Paint booth waste	Water	Additive
0	1	0.77	1.53	1.11	0	0.43	0.72
10	1	0.77	1.53	1.00	0.08	0.43	0.78
20	1	0.77	1.53	0.88	0.15	0.43	0.87
30	1	0.77	1.53	0.77	0.23	0.43	0.65
40	1	0.77	1.53	0.22	0.61	0.43	0.65

**Figure 5:**
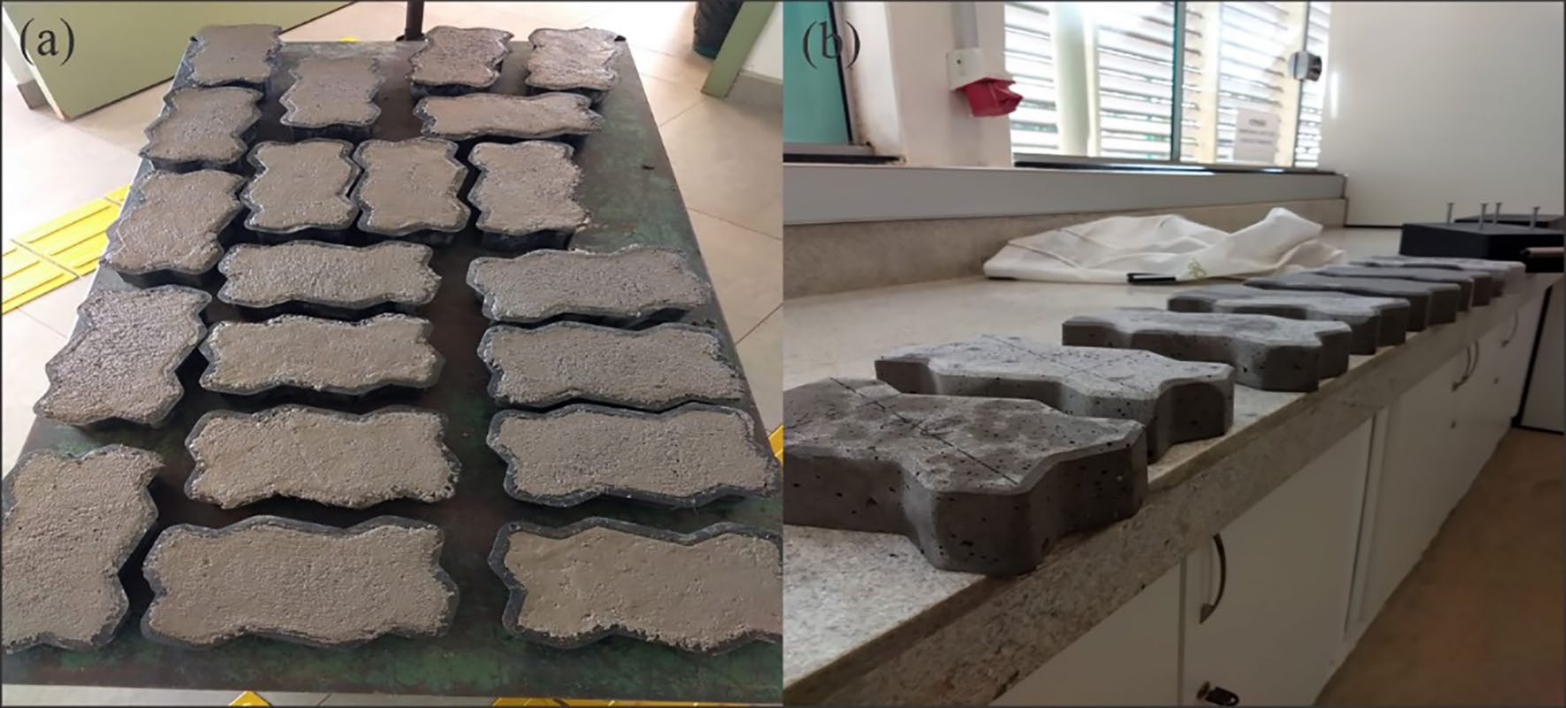
(**a**) Interlocked concrete blocks on vibrating table, (**b**) Unmolded interlocked concrete blocks.

The PBW (with a maximum diameter of 12.5 mm through the sieve) was used as a replacement of 10%, 20%, 30% and 40% in relation to the coarse aggregate mass, however, taking into consideration the difference in the density of the materials. The proportion of water remained the same for all mixtures, changing only the amount of superplasticizer additive. The concrete was mixed in the laboratory and the interlocked concrete blocks were molded into plastic forms with the aid of a vibrating table (Fig. 5). Normally, the concrete block industries use the semi-dry mixture with slump equal to 0, however, due to the method used, the slump of the concrete at 100 ± 25 mm was adopted for better workability.

### Evaluation tests of concrete blocks

#### Concrete consistency

**Figure 6:**
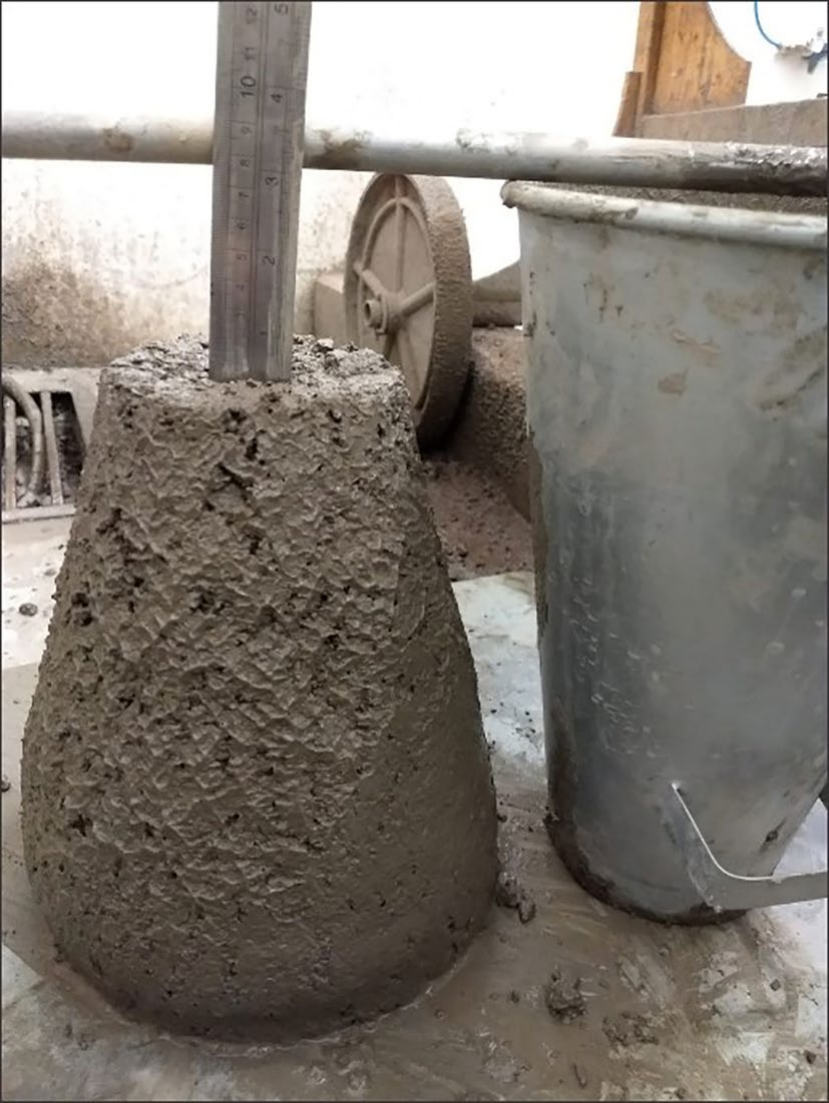
Slump test to evaluate the consistency of the concrete.

The consistency of the concrete mixtures was evaluated using the slump test (ABNT NBR 16,889 2020) (Fig. 6). All concrete mixtures were produced considering a fixed slump of 100 ± 25 mm, defined through preliminary tests with the help of a superplasticizing additive to fix this parameter. Relative air humidity (%) was also evaluated on the days the concrete blocks were molded, based on secondary data. obtained through (INMET 2022).

#### Compressive strength of concrete

For each concrete mixture listed in Table 2, eight interlocked concrete blocks were produced in different curing periods: four for 7 days and four for 28 days, resulting in 40 blocks. The blocks were produced with the following dimensions: 22 cm long, 11 cm wide and 6 cm thick. For the test, a hydraulic press (Intron brand, model PC200CS) with a maximum capacity of 200 t was used. The tests were carried out^26^ and the compressive strength data obtained were subjected to statistical analysis of variance with mean comparison by means of the Tukey test (with a reliability of 95%), in order to verify the influence of the PBW content as a substitute for the coarse aggregate.

#### Water absorption

For the water absorption of the concrete blocks^26^ was four concrete blocks were produced: two blocks for 7 days and two for 28 days of curing. The blocks remained submerged in water for 24 h at a temperature of 23 ± 5 °C. Then, all the parts were dried with the aid of a cloth to determine the saturated surface dry condition (m_sat_). Immediately, the pieces were placed in an oven at 110 ± 5 °C for 24 h. Thus, the dry condition (m_s_) of the concrete blocks was determined. The determination of water absorption is given from Eq. (1). The result of the absorption of the samples is given in (%); where m_sat_ is the mass in saturated condition and dry surface in grams (g) and m_s_ is the mass in dry condition in grams (g).

1 $$A = \frac{{m_{sat} - m_{s} }}{{m_{s} }}$$

#### Microanalysis and environmental performance

The mineralogy, microstructure and environmental performance of the concrete mixtures that presented higher strengths (at 28 days) were analyzed using XRD, and scanning electron microscopy (SEM) techniques, and leaching and solubilization tests, respectively.

The XRD analysis of the concrete mixtures were performed in a similar way to that described in the characterization of materials (item “Paint booth waste”). The SEM technique was performed in a scanning electron microscope (Tescan brand, Vega 3 model), with images in secondary electron mode, electron beam with voltage of 20 kV and energy dispersive spectroscopy (EDS).

The environmental performance was evaluated through leaching tests^27,28^. The NBR 10,005 leaching method^27^ is very similar to the Toxicity Characteristic Leaching Procedure (TCLP—method 1311), the latter being employed to assess the leaching behavior of concrete containing waste materials^29–31^. After 28 days, the specimens were crushed to smaller particles of 9.5 mm. For the leached extract test^27^, the crushed sample was exposed to glacial acetic acid-sodium hydroxide solution (pH ~ 4.93) with a solid/liquid ratio of 1:20. The mixtures were agitated in a rotary shaker at 30 rpm for 18 ± 2 h under 23 ± 2 °C. For the solubilized extract test^28^, the crushed sample was immersed in distilled water with a solid/liquid ratio of 1:4 for 7 days under 23 ± 2 °C.

The leached and solubilized extracts were filtered through a 0.45-μm membrane filter to remove suspended solids. The determination of metals (Ag, Al, As, Ba, Cd, Cr, Cu, Fe, Hg, Mn, Na, Pb, Se, and Zn) present in the liquid extracts was performed by Inductively Coupled Plasma Optical Emission Spectrometry (ICP-OES). Metal concentrations were compared to different standards: annexes F (leached extract) and G (solubilized extract) of the Brazilian standard^7^; CONAMA 460^32^; EPA^33^; Dutch List^34^, and DWQS^35^.

## Results and discussion

### Concrete consistency

**Figure 7:**
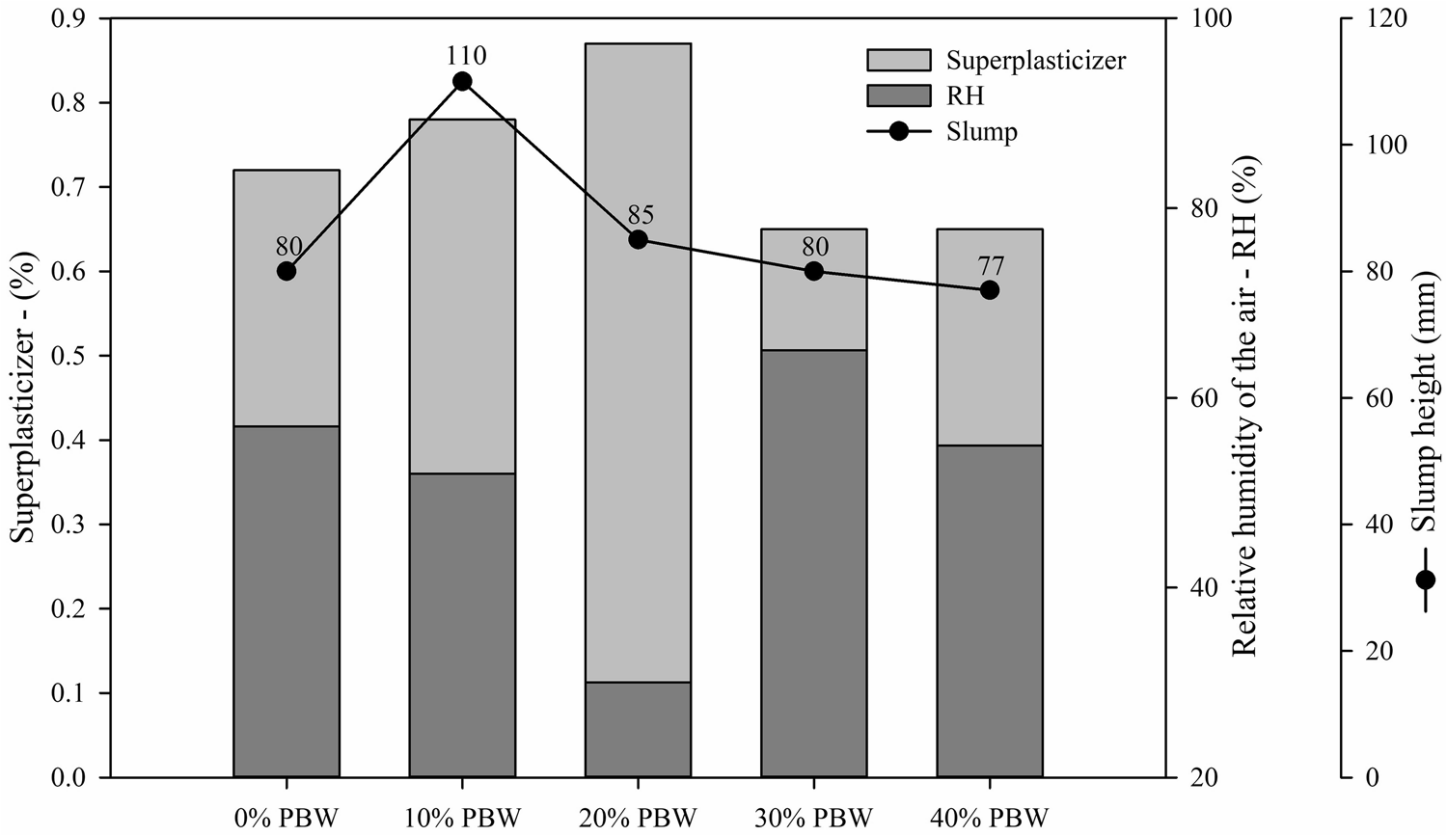
Concrete consistency measured through slump in relation to the superplasticizer additive and to the relative humidity.

Figure 7 shows the abatement values (black points) for all dosages with PBW and their relationship with relative humidity and additive content used. As expected, it was observed that the superplasticizer content (light gray bar) was inversely proportional to the relative humidity content of the air (dark gray bar), i.e., mixtures produced under lower air humidity required a greater amount of additive to achieve the target abatement, as observed mainly in the mixture with 20% PBW.

Although the reduction in workability (slump test) was expected due to the microporous structure of the PBW particles, both the less uniform granulometry and the better packing capacity of the PBW curve (Fig. 3) did not modify this parameter. Furthermore, the shape of the PBW (Table 1) proved to be spherical, which tends to improve workability and the consistency of the concrete mixture, in comparison to conventional aggregate, which has a more lamellar shape^12^.

### Compressive strength

Average results of compressive strength of mixtures with different levels of PBW and curing times are presented in Table 3. The averages were calculated from 4 repetitions in each combination, evaluating the applicability of the waste and the normative values required for interlocked paving^26^. It is noted that the concrete mixtures containing 10% and 20% PBW, at 28 days of curing, reached strengths above 35 MPa, meeting the requirements for light vehicle and pedestrian traffic. Although concrete blocks with 30 and 40% PBW have not reached the strength required for light vehicle traffic, it is still possible to use them in areas without traffic, such as trails, squares, monuments and public parks. Figures 6 and 7 show, respectively, the behavior of compressive strength in relation to PBW contents and curing times. In the figures, the variation ranges refer to the standard deviation, considering the average of all data in the graph for each curing time or PBW content.

**Table 3:** Average values obtained for the different combinations analyzed.

Curing time (days)	PBW content	Average compressive strength (MPa)	Standard deviation (MPa)	Coefficient of variation (%)
7	0	37.5	2.0	5.0
	10	29.5	1.9	6.0
	20	29.3	2.6	8.2
	30	27.0	2.0	6.9
	40	25.6	1.7	6.3
28	0	37.5	2.9	7.3
	10	35.1	2.0	5.5
	20	36.9	1.9	4.9
	30	34.4	0.5	1.4
	40	30.1	1.2	3.7

**Figure 8:**
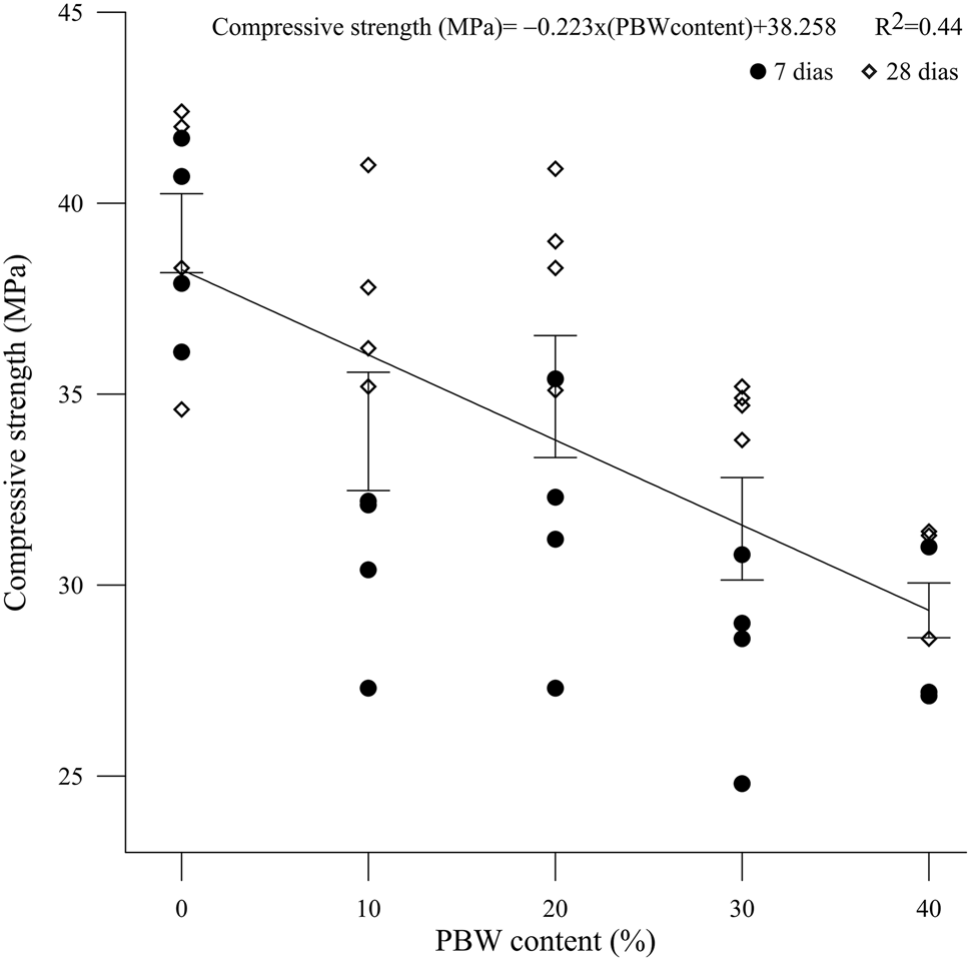
Compressive strength behavior versus PBW content.

In Fig. 8 it is possible to observe a drop in strength with the increase in the replacement of coarse aggregate by PBW, possibly due to the waste increasing the porosity of the concrete matrix. In case of concrete mixtures containing 10% and 20% PBW, at 28 days of curing that kept strengths above 35 MPa, there was no greater drop possibly due to the packing effect of the PBW, which has a more distributed grain size than the coarse aggregate (see Fig. 3).

**Figure 9:**
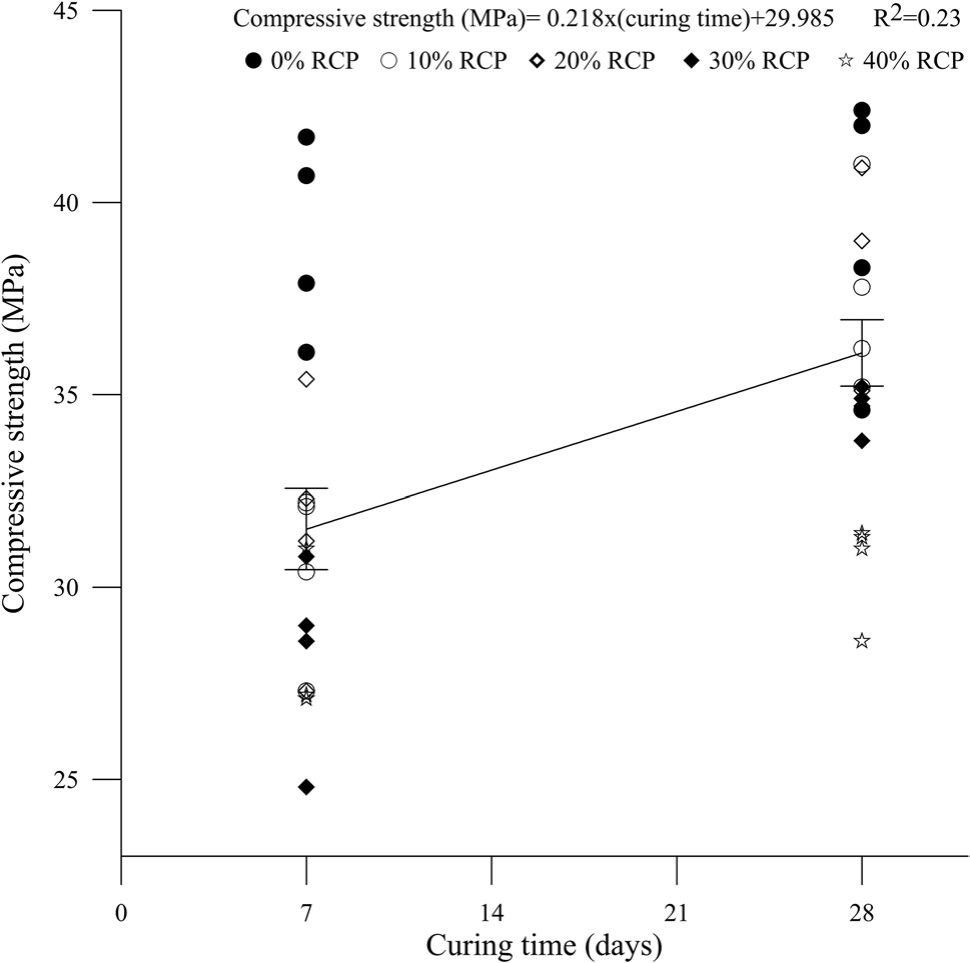
Compressive strength versus curing time.

The curing time influenced the strength of concrete mixtures containing PBW (Fig. 9), since for the concrete with 0% of replacement content, for 7 and 28 days, the strength of 37.5 MPa was maintained due to the type of cement used. For 10%, 20%, 30% and 40% there was a variation of 18.98%, 25.94%, 27.40% and 17.58% respectively, from 7 to 28 days. This possibly occurred due to the structure of the concrete matrix, PBW being more porous, where voids were filled by the cementing gel during the curing process. Another possibility is that it may be related to the presence of polymers and calcium-based materials present in concrete, which cause expansion of the mortar and contribute to filling the concrete matrix over the curing time.

For mixtures with contents of up to 30% of coarse aggregate replacement by PBW, there was a small drop in the compressive strength of the interlocking concrete blocks, after 28 days of curing. Comparing them to the reference blocks (0% PBW), the mixtures with 10, 20 and 30% obtained an average reduction of 6.4%, 1.6% and 8.26%, respectively. This reduction occurs because PBW causes an expansion in the mortar mixture due to the presence of polymers and calcium-based materials. These components of industrial/automotive paint paints, in addition to acting as a filler in the matrix, also produce a structure with small amounts of silica and alumina tetrahedrons, which can form small crystals, as recognized by previous studies^5,8,36^.

Similar results were verified^11^ where it was found that the incorporation of 20% automotive paint waste in bituminous mixtures did not cause significant changes in the mechanical properties, compared to the reference mixtures. Also, considering that industrial paint waste has a porous microstructure^5,10^, the mixture with a content of 40% PBW reduced the average strength of 19.73%, when compared with mixing (0% PBW) possibly caused by voids in the concrete matrix.

Tables 4 and 5 present the result of the statistical analysis of variance with comparison of means, using the Tukey test, considering a significance level of 95%. Table 4 shows that the drop in strength with the increase in PBW content was not very expressive when comparing concrete blocks with 10, 20 and 30% PBW to the reference (0% PBW) one. This is because blocks with 10, 20 and 30% PBW presented statistically equal means of compressive strength (*P* value < 0.001). When comparing the concrete block with 40% PBW replacement to the reference (0% PBW), there is a statistically significant difference of 25.17%.

**Table 4:** Tukey’s multiple comparison for compressive strength × PBW content.

PBW content	Average compressive strength (MPa)
0	39.21 a
10	34.02 b
20	34.94 b
30	31.47 bc
40	29.34 c

Means that do not share a letter are statistically distinct.

**Table 5:** Tukey’s multiple comparison for compressive strength × curing time.

Curing time (days)	Average compressive strength (MPa)
7	31.51 a
28	36.1 b

Means that do not share a letter are statistically distinct.

Regarding the influence of curing time (Table 5), there is a significant difference (*P* value < 0.001) between the average values for 7 and 28 days. The curing time was a factor that positively influenced the strength of the concrete, generating an average increase of 14.57% from 7 to 28 days of curing, statistically corroborating the results observed in Fig. 9.

**Figure 10:**
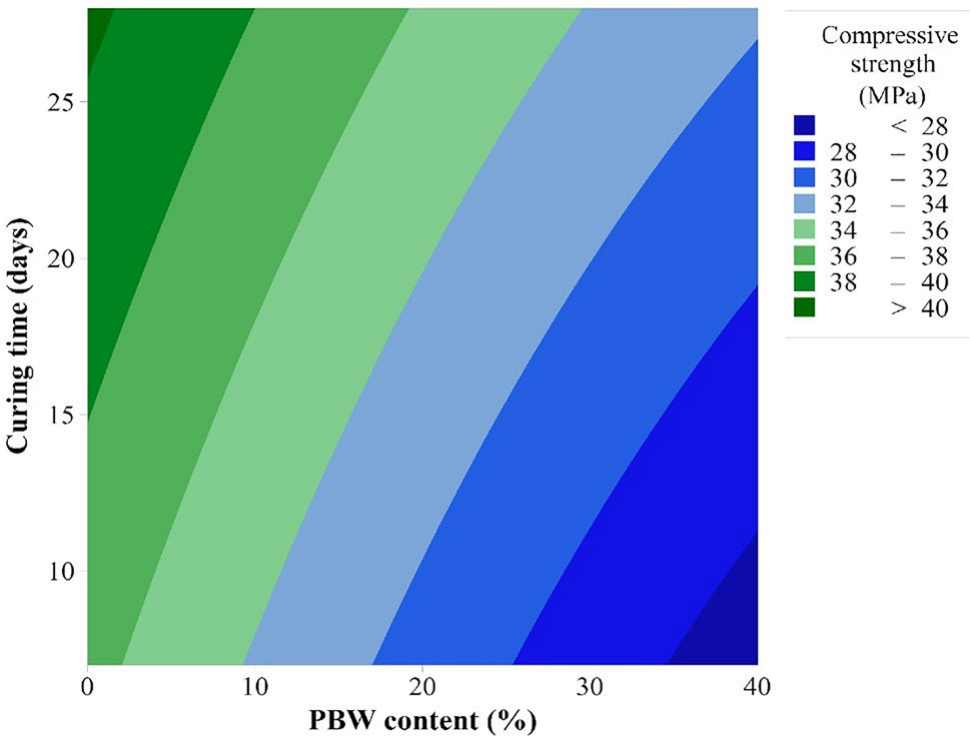
Contour plot of the compressive strength as a function of curing time and PBW content.

In Fig. 10 the contour surface with compressive strength behavior with both factors (curing time and PBW content can be observed). After statistical analysis of variance with a generic linear model, no predominance of interactions between the PBW content and the curing time was found, allowing us to infer that, in isolation, lower levels of PBW and longer curing times result in higher average strengths (39.21 MPa).

### Water absorption

**Figure 11:**
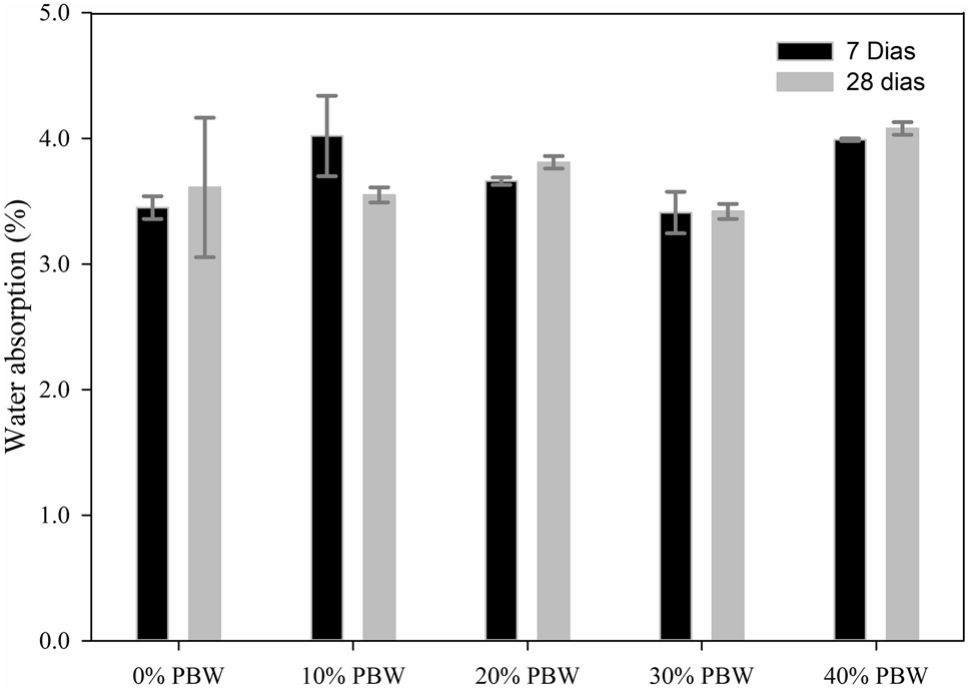
Water absorption for concrete blocks as a function of different curing times and PBW content.

According to standard, the average value of water absorption of concrete must be less than or equal to 6% and no individual value must be greater than 7%^26^. All concrete mixtures with different substitutions of coarse aggregate by PBW showed average values of water absorption below 4.08%, meeting the aforementioned Brazilian standard (Fig. 11). Still in Fig. 9 it is observed that the curing time did not influence the water absorption. Larger amounts of PBW in the mixtures tend to cause greater water absorption, possibly because the incorporation of PBW increases the porosity of the concrete matrix.

### X-ray diffraction (XRD)

**Figure 12:**
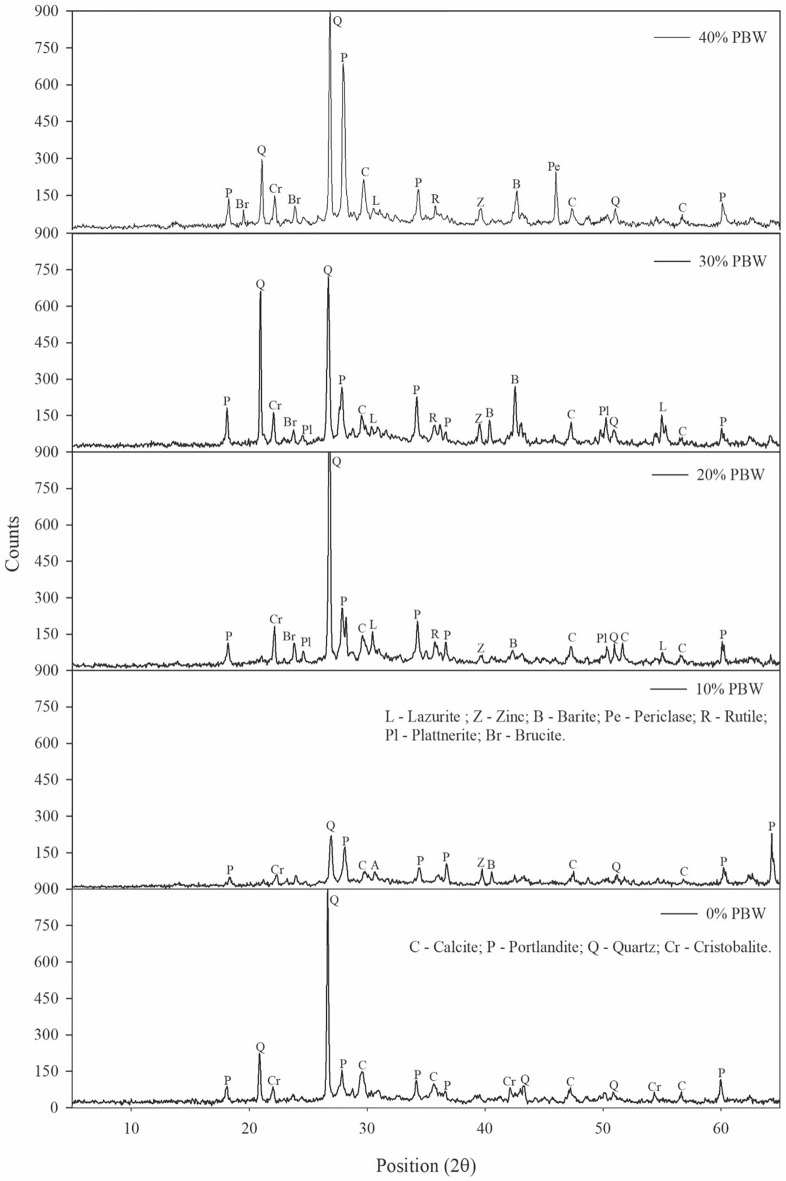
Diffractograms of samples of concrete blocks with different PBW contents.

Figure 12 shows the diffractograms of the concrete blocks with PBW (10, 20, 30, 40% PBW) and the reference material (0% PBW), at 28 days of curing. The samples showed a mineralogy composed of semi-crystalline and crystalline phases, which share the presence of calcite (CaCO_3_), quartz (SiO_2_) and cristobalite (SiO_2_) from concrete materials. Portlandite (Ca(OH)_2_), a mineral present in all the samples, is formed in the hydration reactions of Portland cement. It can also be observed the presence of zinc (Zn) and Barite (Ba(SO)_4_) in all the samples with PBW; such minerals come from the waste (Fig. 3b). It is also observed that as the percentage of PBW increases in the concrete mixture, new phases are identified, such as utile (TiO_2_), plattnerite (PbO_2_) and lazurite (Na_3_CaAl_3_Si_3_O12S), as well as periclase (MgO) and brucite (Mg(OH)_2_).

Due to the chemical reactions that occur in concrete, the PWB is exposed to an alkaline attack, which raises the pH of the means and promotes the dissolution of zinc, calcium and magnesium. Such a process exposes new mineral phases of rutile, plattnerite and lazurite, which are found in white, black and blue pigments for paints respectively, as recognized by other studies^37–40^. These new phases arising from the pigmentation of paints, but not observed in the PBW diffractogram (Fig. 4), suggest that this waste is a material with a highly heterogeneous composition due to the different pigments used in the industrial paint booth.

### Scanning electron microscopy (SEM)

Analysis of the microstructure of the concrete blocks with the addition of 20% and 40% PBW were carried out, and these contents were adopted due to the interest in observing the effect of the intermediate and maximum substitutions used.

**Figure 13:**
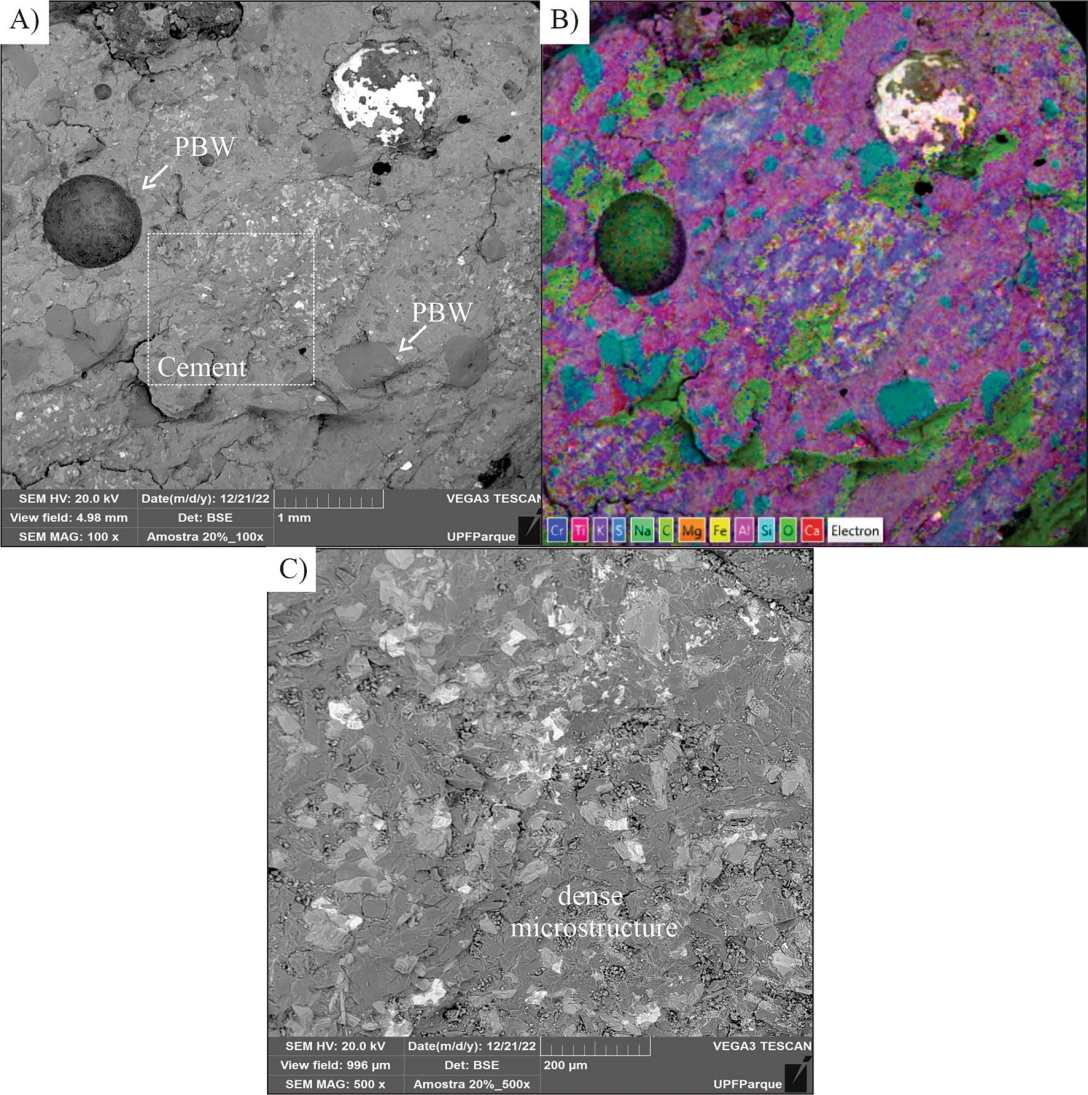
SEM Images of (**a**) Concrete with 20% PBW—100 × magnification, (**b**) Distribution of concrete elements with 20% PBW—100 × magnification, (**c**) SEM Images of Concrete with 20% PBW—500 × magnification.

**Figure 14:**
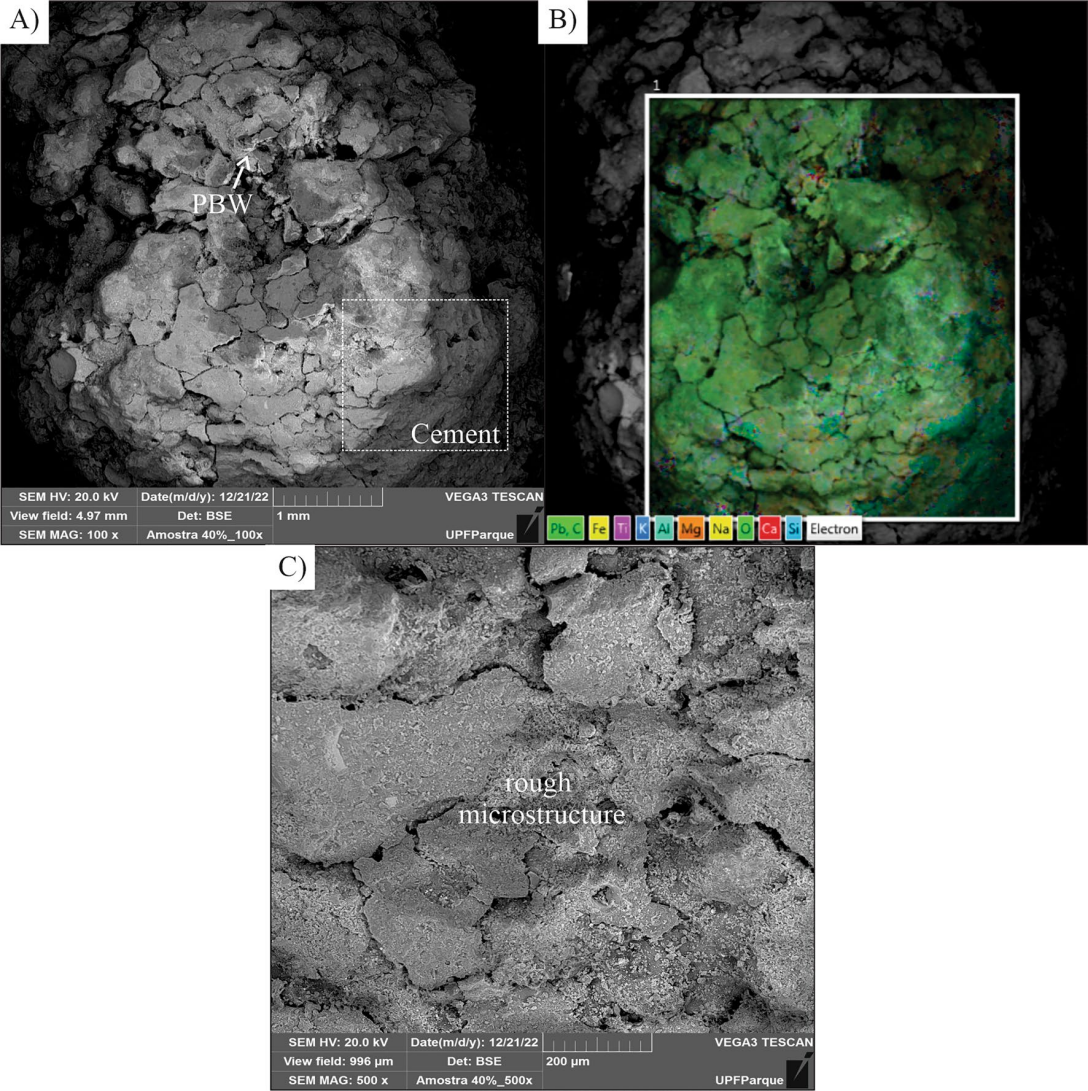
SEM Images of (**a**) Concrete with 40% PBW—100 × magnification, (**b**) Distribution of concrete elements with 40% PBW—100 × magnification, (**c**) SEM Images of Concrete with 40% PBW—500 × magnification.

In Fig. 13a–c, it is noted that the mixture containing 20% PBW is denser and with better homogeneity, corroborating the greater gains in compressive strength results. The mixture with 40% PBW (Fig. 14a–c) presents a rougher characteristic and a greater amount of micropores and microcracks, represented by the dark regions, justifying the lower strength and higher water absorption.

The element distribution image (EDS) of the mixtures containing both 20% PBW (Fig. 13b) and 40% PBW (Fig. 14b) showed that the PBW was incorporated in a very diffuse way in the concrete matrix, interpreted by the areas rich in titanium and lead (chemical characterization of PBW and new phases of mixtures—Fig. 11) and showed isolated presence some particles of PBW and other metals as chromium for 20% PBW (Fig. 12b).

### Environmental performance

Tables 6 and 7 show the results of the leached and solubilized extracts, respectively, from concrete mixtures with 10, 20, 30 and 40% PBW and from the raw waste, compared with the maximum limits (VROM, 2000; MHLW, 2003; ABNT NBR 10,004 2004; EPA 2021). The leached extracts of all concrete mixtures with PBW showed metal concentrations lower than the limits of annex F of the Brazilian standard^7^, indicating that these materials entails no environmental risk in terms of metal toxicity. All concrete-PBW mixtures leached Ba and Cr concentrations above the limit of at least one water quality standard. Cr leaching comes from high initial strength Portland cement as traditional binder raw materials have this element in their chemical compositions^41^. Furthermore, in the extracts leached from the mixtures with PBW, there was a reduction in Ba concentrations, compared to the raw waste, denoting the encapsulation/immobilization capacity of metals in the concrete matrix. For the 20% PBW mixture (the mixture presented satisfactory mechanical performance for interlocking concrete blocks), the reduction of Ba concentration in the leached extracts was 86.4%.

**Table 6:** Chemical analysis of leached extracts from gross PBW and concrete mixtures with 10, 20, 30, and 40% PBW (mg L^−1^).

Metal	Raw waste	Concrete with PBW				Standards				
		10%	20%	30%	40%	NBR 10,004 (annex F)	CONAMA 460^b^	EPA^c^	Dutch List^d^	DWQS-Japan^e^
As	–^a^	–^a^	–^a^	–^a^	–^a^	1.0	0.01	0.01	0.01	0.01
Ba	**3.3**	**0.1**	**0.5**	**0.8**	**0.4**	70.0	**0.7**	**2.0**	**0.05**	**–**
Cd	–^a^	–^a^	–^a^	–^a^	–^a^	0.5	0.005	0.005	0.0004	0.01
Pb	–^a^	–^a^	–^a^	–^a^	–^a^	1.0	0.01	0.015	0.015	0.01
Cr	–^a^	**0.1**	**0.1**	**0.1**	**0.2**	1.0	**0.05**	**0.1**	**0.001**	**0.05**
Hg	–^a^	–^a^	–^a^	–^a^	–^a^	0.1	0.001	0.002	0.00005	0.0005
Ag	–^a^	–^a^	–^a^	–^a^	–^a^	5.0	0.05	–	–	–
Se	–^a^	–^a^	–^a^	–^a^	–^a^	1.0	0.01	0.05	–	0.01

^a^Below detection limit.

^b^Guiding values for groundwater.

^c^Maximum contaminant levels—national primary drinking water regulations.

^d^Groundwater target values.

^e^Drinking water quality standards.

Values in bold represent metal concentrations that surpassed the limits of one or more standards.

**Table 7:** Chemical analysis of solubilized extracts from gross PBW and concrete mixtures with 10, 20, 30, and 40% de PBW (mg L^−1^).

Metal	Raw waste	Concrete with PBW				Standards				
		10%	20%	30%	40%	NBR 10,004 (annex G)	CONAMA 460^b^	EPA^c^	Dutch List^d^	DWQS-Japan^e^
Al	**1.0**	0.17	–^a^	**0.4**	**0.35**	**0.2**	3.5	–	–	**0.2**
As	–^a^	–^a^	–^a^	–^a^	–^a^	0.01	0.01	0.01	0.01	0.01
Ba	**0.9**	**1.35**	**2.16**	**1.16**	**1.28**	**0.7**	**0.7**	2.0	**0.05**	–
Cd	–^a^	–^a^	–^a^	–^a^	–^a^	0.005	0.005	0.005	0.0004	0.01
Pb	–^a^	–^a^	–^a^	–^a^	–^a^	0.01	0.01	0.015	0.015	0.01
Cu	–^a^	–^a^	–^a^	–^a^	–^a^	2.0	2.0	1.3	0.015	1.0
Cr	–^a^	0.01	0.01	0.01	0.03	0.05	0.05	0.1	**0.001**	0.05
Fe	**2.5**	–^a^	–^a^	–^a^	–^a^	**0.3**	**2.45**	–	–	**0.3**
Mn	**0.2**	–^a^	–^a^	–^a^	–^a^	**0.1**	0.4	–	–	**0.05**
Hg	–^a^	–^a^	–^a^	–^a^	–^a^	0.001	0.001	0.002	0.00005	0.0005
Ag	–^a^	–^a^	–^a^	–^a^	–^a^	0.05	0.05	–	–	–
Se	–^a^	–^a^	–^a^	–^a^	–^a^	0.01	0.01	0.05	–	0.01
Zn	**6.7**	–^a^	–^a^	–^a^	–^a^	**5.0**	**1.05**	–	**0.065**	**1.0**
Na	1.7	9.74	9.72	9.33	7.70	200	–	–	–	200

^a^Below detection limit.

^b^Guiding values for groundwater.

^c^Maximum contaminant levels—national primary drinking water regulations.

^d^Groundwater target values.

^e^Drinking water quality standards.

Values in bold represent metal concentrations that surpassed the limits of one or more standards.

In the gross PBW there was the solubilization of Al, Ba, Fe, Mn, and Zn in concentrations above the limit of Brazilian standard^7^. In the concrete mixtures with PBW, concentrations of Fe, Mn, and Zn were not detected in the solubilized extracts. The concrete blocks with PBW solubilized concentrations of Ba higher than that observed in the solubilized extract of the gross waste. This is justified by the fact that barite (barium sulfate present in the PBW) is a mineral that has good solubility in alkaline environments [pH 11–12]^42^, as found in concrete mixtures with waste. This is because the solubilized extracts of concrete with 10, 20, 30 and 40% PBW showed average pH values of 12.8. Regarding the limit of the water quality standards (VROM, 2000; MHLW, 2003; EPA, 2021), Ba solubilization occurred in all concrete-PBW mixtures possibly due to the high pH of the medium (13.12). Despite the solubilization of Ba in the solubilized extracts, the water contamination by this chemical element is low, which does not compromise the future use of mixtures with 20% PBW which showed better mechanical performance.

## Conclusion

Based on the results obtained, the following conclusions were drawn:

- The PBW did not influence the results of consistency of the concrete in the fresh state, since the mixtures of 0 and 40% with PBW obtained similar slump results;
- The compressive strength of interlocking concrete blocks tends to reduce with the addition of PBW. The strength gains of the mixtures were influenced by the curing time of the concrete with PBW, making it possible to reach 36.9 MPa after 28 days in the mixture with 20% PBW;
- The concrete blocks with higher amounts of PBW showed higher water absorption due to the greater presence of micropores;
- In the mixtures with PBW substitution, new phases were identified, such as rutile, plattnerite, lazurite, periclase, and brucite from the heterogeneous composition of the waste;
- The distribution of the elements showed that the PBW was incorporated in a very diffuse way into the concrete matrix, with punctual concentrations of Fe, areas rich in titanium, chromium and lead in addition to silicon and calcium;
- The incorporation of PBW in the concrete proved to be an interesting alternative to encapsulate Ba and eliminate the leaching of metals from the waste as showed no environmental risk in terms of metal toxicity;
- Mixtures with up to 20% replacement of coarse aggregate by the PBW showed satisfactory mechanical and environmental performance to be applied in interlocked concrete blocks.

## Data Availability

The datasets used and/or analyzed during the current study are available from the corresponding author on reasonable request.
